# ﻿Ethogram and classification of the mating and egg-laying behaviour of the Southeast Asian apple snail *Pilavirescens* (Deshayes, 1824) (Mollusca, Gastropoda, Caenogastropoda, Ampullariidae)

**DOI:** 10.3897/zookeys.1180.106498

**Published:** 2023-09-26

**Authors:** Supunya Annate, Ting Hui Ng, Chirasak Sutcharit, Somsak Panha

**Affiliations:** 1 Animal Systematics Research Unit, Department of Biology, Faculty of Science, Chulalongkorn University, 254 Phayathai Road, Pathumwan, Bangkok 10330, Thailand Chulalongkorn University Bangkok Thailand; 2 Institute for Tropical Biology and Conservation, Universiti Malaysia Sabah, Jalan UMS, 88450 Kota Kinabalu, Sabah, Malaysia Universiti Malaysia Sabah Sabah Malaysia; 3 Academy of Science, The Royal Society of Thailand, Bangkok 10300, Thailand Academy of Science, The Royal Society of Thailand Bangkok Thailand

**Keywords:** Ampullariidae, apple snails, behavioural observation, copulation, *Pila*, reproductive biology, Southeast Asia, spawning

## Abstract

The status of the indigenous Southeast Asian apple snails belonging to the genus *Pila* is of concern due to their fast rate of population decline, possibly as a result of multiple factors including habitat loss or disturbance and the introduction of globally-invasive apple snails, *Pomacea* spp. Conservation actions, including captive breeding of the native *Pila* species, have been suggested as urgent remedial practices, but the lack of knowledge regarding the fundamental reproductive biology of indigenous *Pila* spp. makes such practices difficult. In the present study, observations on the mating and egg-laying behaviour of an economic valuable apple snail native to Southeast Asia, *P.virescens*, were conducted using video recording to examine and describe their reproductive behaviour under a laboratory condition. A total of 15 types of mating and seven egg-laying behaviour were recorded. The mating sequence which subsequently resulted in egg laying was comprised of seven types of major sequential behaviour: mate probing, mounting, shell circling, positioning, insemination posture, sheath withdrawal and dismounting. Rejection of mating attempts by the female was frequently observed. Egg laying occurred during either day or night. A sequence of seven distinct types of behaviour were performed during oviposition: climbing, positioning, forming a temporary tube, mucous secreting, egg depositing, leaving and resting. Overall, these results provide an understanding of the egg-laying behavioural process and highlight its complexity in *P.virescens*. In addition, detailed ethograms of mating and egg-laying behaviour were derived. These will promote further systematic comparative studies of the reproductive behaviour of apple snails.

## ﻿Introduction

The Southeast Asian apple snail *Pilavirescens* (Dehayes, 1824) is distributed in several countries of Southeast Asia, including Thailand, Vietnam, Cambodia, Laos, Malaysia, Indonesia, Myanmar and Philippines (Keawjam 1986, 1990; Cowie 2015; Ngor et al. 2018; Ng et al. 2020). Historically, *P.virescens* was reported to occur in various freshwater habitat types, both standing and moving water bodies (as *Pilapolita* in Keawjam (1986, 1990); Thaewnon-Ngiw et al. (2003a, b)). However, in the last few decades, many populations of *P.virescens* have declined and disappeared from where they were formerly known to be present (Ng et al. 2020). On the other hand, those habitats have often been invaded by apple snails from the genus *Pomacea* (Ng et al. 2020), two species of which have invaded globally, including in Thailand (Cowie et al. 2012). Moreover, in some areas, populations of *P.virescens* have entirely been replaced by *Pomacea* snails (Marwoto et al. 2020; Ng et al. 2020).

Although the direct impact of *Pomacea* snails on native *Pila* snails is unknown, many studies have indicated that populations of *Pila* spp. decline after the introduction of *Pomacea* spp. (Chaichana and Sumpan 2014; Ng et al. 2019, 2020; Marwoto et al. 2020). Habitat loss or disturbance and pollution, for instance, the use of molluscicides to remove *Pomacea* snails, may be additional factors that threaten native *Pila* species (Ng et al. 2020). Therefore, *P.virescens* is under strong threat. Although *P.virescens* is categorised in the ‘Least Concern’ category by the IUCN, this assessment was based on decades-old data that require updating (as *Pilapolita* in Madhyastha and Sri-aroon (2012)). In contrast, more recent data suggests that *P.virescens* may be in risk of extinction (Ngor et al. 2018; Marwoto et al. 2020; Ng et al. 2020) and deserves urgent conservation.

Ng et al. (2020) suggested captive breeding of native *Pila* species from different localities should be encouraged in order to enhance conservation of wild populations as they are consumed widely in Southeast Asia, especially for *P.virescens* in Thailand (Keawjam 1986; Ng et al. 2020), where it is known as hoy pang. Such aquaculture by captive breeding may help decrease overharvesting of wild populations. However, culturing *P.virescens* is difficult (Keawjam 1986). The lack of knowledge on its reproductive biology is an important obstacle to captive breeding. Understanding the reproductive behaviour of this species would contribute to resolution of practical difficulties of captive breeding and conservation of *P.virescens*.

There have been several studies on the apple snail genus *Pila* in Southeast Asia, most of which mainly focused on the taxonomy of this group (Annandale 1920; Keawjam 1986, 1987a, b, 1990; Thaewnon-Ngiw et al. 2003a, 2004; Low et al. 2013; Tan et al. 2013; Ng et al. 2014, 2020; Cowie 2015), parasitology (Punyagupta 1965; Tesana et al. 2008; Komalamisra et al. 2009) and ecology (Chaichana and Sumpan 2014, 2015; Pradabphetrat et al. 2017; Ng et al. 2019). However, few details on their reproductive biology have been studied. The morphology and anatomy of the reproductive system and egg mass characteristics of five *Pila* species: *P.celebensis*, *P.gracilis*, *P.pesmei*, *P.turbinis* and *P.virescens* that are distributed in Thailand have been described (Keawjam 1987a; Ng et al. 2020). Egg mass characteristics, clutch size, incubation period and reproductive seasons were reported in *P.celebensis* (as *P.ampullacea* in Djajasasmita (1987)) and *P.pesmei* (Lamkom and Phosri 2017). For Southeast Asian apple snails, mating behaviour is only known for *P.turbinis* (as *P.ampullacea* in Panha (1985)). Egg and egg mass characteristics of *P.virescens* in the Philippines were described more than a century ago (Semper 1862). Thus, the biology, ecology and behaviour of reproduction of *Pila* in Southeast Asia, which are crucial to understand the evolution of diversity in apple snails (Hayes et al. 2009), mostly remain to be investigated. The aims of this study were to describe the mating and egg-laying behaviour of *P.virescens*, with a detailed ethogram of mating and egg-laying behaviour for future comparative studies amongst apple snails.

## ﻿Methods

### ﻿Rearing

The descendants of nine juvenile snails of *P.virescens* bought from a farmer in Nong Song Hong, Ban Paeow, Samut Sakhon, Thailand in October 2017 were used in this study. The nine juveniles were transferred to the Animal Systematics Research Unit, Department of Biology, Faculty of Science, Chulalongkorn University, Bangkok, Thailand. They were reared in a 136 l concrete container containing 80 l tap water (20 cm depth) at ambient temperature under a natural photoperiod until mature. They were fed with lettuce *ad libitum*. Water changing and cleaning of the concrete container was performed every two weeks. Females laid egg masses on the wall of the concrete container in May 2018. Egg masses were gently removed from the wall and incubated at 29 ± 2 °C under a 12:12 h (light/dark) photoperiod until hatching. Hatchlings were reared in 27 l glass aquaria (10 snails/aquarium) with 15 l tap water (17 cm depth) at room temperature under a 12:12 h (light/dark) photoperiod and fed with lettuce *ad libitum* until three-months old. Water was changed and aquaria were cleaned weekly.

When the hatchlings reached three months old, they were sexed by placing them on a soft cloth in a position (aperture on top) such that their penis sheath could be observed directly when the snails opened their aperture and tried to pull their body to a normal position. The sexed snails were transferred to 136 l concrete containers (20 snails/container) and reared in the same condition as their parents. Males and females were reared separately in different containers.

For confirmation of sexual maturity after snails reached six months old, 10 males and 10 females were randomly chosen from rearing containers and placed into a collective concrete container, where mating and egg mass production events were observed daily. Based on the production of egg mass, it was confirmed that snails become sexually mature within six months after hatching. Based on this, all individuals used for the behaviour study were older than six months old and, thus, judged to be mature.

### ﻿Mating behaviour recording

**Figure 1.**
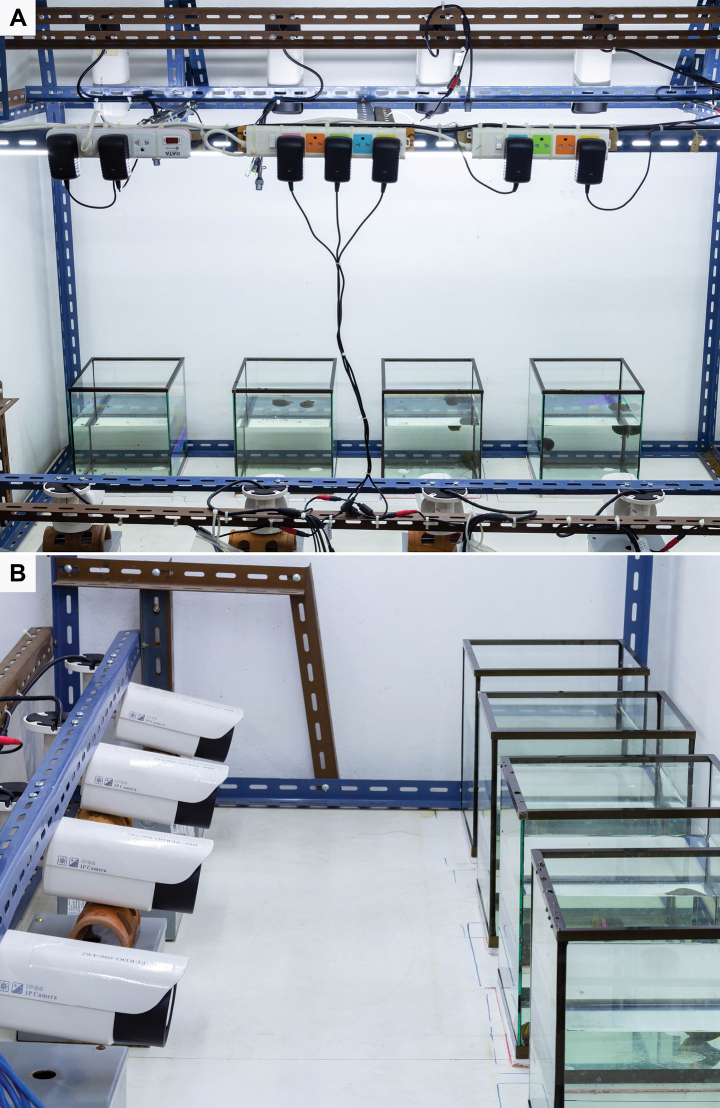
The (**A**) top and (**B**) lateral view of the experimental setup for observing the mating behaviour.

In order to avoid learning effects, virgin mature snails were used to examine mating behaviour (Trigwell et al. 1997). A total 78 pairs of virgin males and females were selected for observation of mating behaviour. Their behaviour was recorded using infrared digital video cameras (5 MP HD IP Camera, Model YM500L). Each pair was kept in a 17.5 l glass aquarium containing 10 l of tap water (16 cm depth) at room temperature (27 ± 2 °C) under a 12:12 (light/dark) photoperiod. Two video cameras were used for each aquarium, one recording the top view and the other the lateral view. The experimental set-up for this video is shown in Fig. 1. Three aquaria, each with one pair, were concurrently recorded in each trial and a total of 26 trials were conducted to examine the behaviour of the 78 pairs.

Male and female snails were marked with nail polish on the shell, the former with two stripes of blue colour and the latter with a pink stripe, in order to differentiate them in the video recordings by colour and the number of stripes (for night activity). Marked snails were then transferred to 27 l glass aquaria (15 l tap water to a depth of 17 cm) and males and females were maintained separately for 24 h to acclimatise them. After this procedure, males with shell lengths from 35 to 55 mm and mean 44.38 ± S.D. 2.98 mm and females with shell lengths from 40 to 56 mm and mean 49.65 ± S.D. 3.07 mm were randomly paired in the observational aquaria and their behaviour was recorded continuously for 24 h.

In seven of the 78 observed pairs, video recordings were incomplete due to electrical problems. In addition, nine of the remaining pairs did not show any behavioural interaction between the two snails for three hours. Thus, these 16 pairs were excluded from the analysis. Consequently, video recordings of 62 pairs were used for behavioural data collection and analysis.

### ﻿Egg-laying behaviour recording

**Figure 2.**
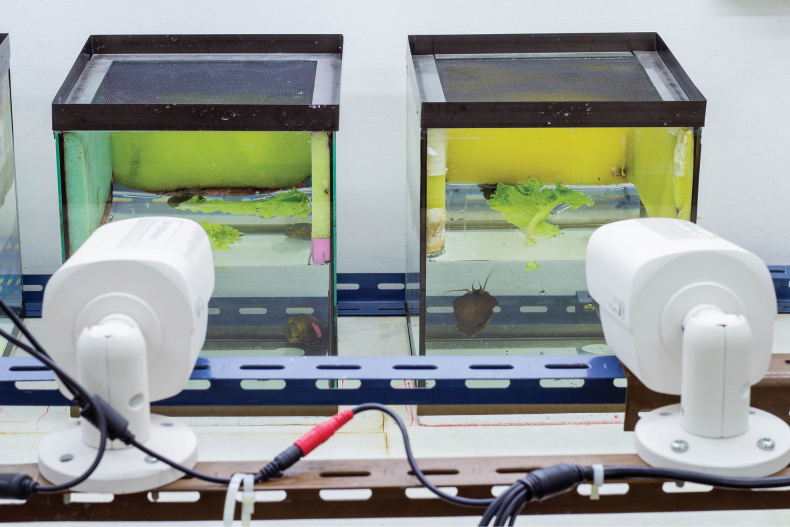
Experimental setup for egg-laying behaviour observation.

Egg-laying behaviour was video-recorded using the same camera model as used in the mating behaviour recording, but with a different experimental setup (Fig. 2). Sponge wipes were attached to the three sides of an aquarium wall (above water) to prevent the snail from laying eggs and to provide the open side without a sponge wipe as the egg-laying area (260 cm^2^). The egg-laying aquarium was filled with 10 l of tap water (16 cm depth). An infrared digital camera was installed in front of the egg-laying area.

Females that mated with males in the mating behaviour recording aquaria (20 snails) and in the collective container (28 snails) were numbered using nail polish and released into the egg-laying aquaria (2–4 mated females per aquarium) with 10 l tap water. Types of behaviour were recorded simultaneously in four egg-laying aquaria over a period of 60 days continuously or until egg laying occurred. The observation was performed at room temperature (27 ± 2 °C) under a 12:12 (light/dark) photoperiod. Water was changed and the aquaria were cleaned weekly. Snails were fed with fresh lettuce *ad libitum* throughout the observation period.

A total of 15 females laid eggs. Five of them laid eggs out of the camera view due to the detachment of spongy wipe from the aquarium wall allowing snails to lay eggs on the other sides of aquaria. Therefore, 10 egg-laying occasions by 10 females (five females from the mating behaviour recording and five females from the collective aquaria) were analysed in this study.

### ﻿Mating behaviour examination

Types of behaviour were examined by watching the video recordings at 1×–8× the normal speed using the GOM player software (GOM & COMPANY). In order to list all behavioural occurrences with their entire durations and frequencies, continuous sampling was used for behavioural analysis. An ethogram of behaviour during the mating process, which included both general and mate-related behaviour, was constructed. Some types of behaviour and behavioural definitions that have been previously reported in other freshwater snails, such as *Pomaceacanaliculata* (Burela and Martín 2009), *Biomphalariaglabrata* (Trigwell et al. 1997) and planorbid snails (Soldatenko and Petrov 2012), were applied to this ethogram construction.

### ﻿Egg-laying behaviour examination

All the occurrences, duration and frequency of each type of behaviour of females during the entire egg-laying period—from moving above water until finishing egg laying and moving back to the water—were examined. We examined their behaviour for 4 h before and 4 h after the egg-laying process in order to examine if females showed any specific pattern of behaviour before laying eggs or after laying eggs.

### ﻿Data analysis

The duration of each type of behaviour was quantified in minutes and presented as the mean ± one standard deviation (SD), while the frequency of each type of behaviour was calculated as number of occurrences per entire duration of the phase, in which the behavioural event occurred.

## ﻿Results

### ﻿Mating behaviour

The observation revealed a total of 19 types of behaviour (showed in Table 1): four general behavioural and 15 mating behavioural types. Mating behaviour patterns could be classified following the success of mating process into two different patterns: complete and incomplete mating processes.

**Table 1.** Ethogram of mating behaviour.

Behaviour	Code	Description
**General behaviour**		
Breathing	Bt	Gas exchange at water surface by opening siphon directly to air.
Moving	Mo	Walking, swimming or climbing around aquarium.
Resting	Rt	Motionless with coiled tentacles.
Sheltering	St	Completely close aperture with operculum.
**Mating behaviour**		
Mate probing	Mp	Contacts with cephalic tentacles, labial palps or feet.
Mounting	Mt	Snail mounts on another snail with the foot having completely lost its hold on the aquarium.
Shell circling	Sc	Snail claws another snail’s shell, moves over it in either a clockwise or counter-clockwise direction.
Positioning	Pt	Male adheres on the female’s last whorl at the right side of the shell rim above the opening of the female gonopore.
Insemination posture	Ip	Male tightly adheres to the female’s shell with cephalic tentacles coiled; female is usually motionless, but occasionally moves around.
Sheath withdrawal	Swd	Male withdraws penis sheath from female gonopore: recognised from the first movement of the male foot and tentacles after long passive stage during insemination posture.
Dismounting	Dmt	Male detaches from the female’s shell.
Passive	Ps	Motionless, while copulation with tentacles coiled and foot contracted; male firmly adheres to female shell; female firmly adheres to aquarium or detaches from aquarium.
Mate guarding	Mg	Male mounts on female shell after sheath withdrawal.
Operculum closing	Oc	Imminently closes aperture with operculum responding to any contacts from another snail.
Retracting	Rtt	Contracts cephalopodium into shell, tightly adheres to aquarium or completely detaches from the aquarium.
Jerking	Jk	Contracts and releases cephalopodium into- and out of shell several times.
Swinging	Sw	Rotates shell several times in a counter- and clockwise way.
Withdrawal-wrestling	Ww	Female pushes male shell with the operculum.
Shell pushing	Sp	Male pushes female by moving its shell.

**Figure 3.**
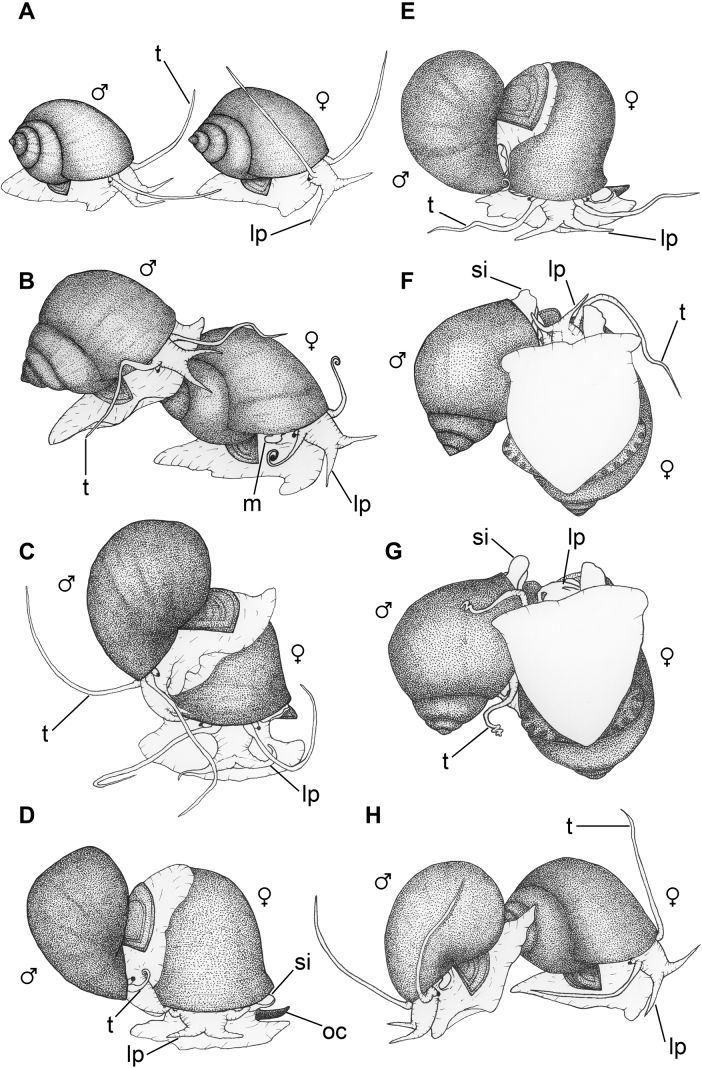
Schemes of mating behaviour in *P.virescens***A** mate probing **B** mounting **C** shell circling **D** positioning **E** insemination posture (lateral view) **F** insemination posture (ventral view) **G** sheath withdrawal **H** dismounting. Abbreviations: m, mantle; lp, labial palps; oc, operculum; si, siphon; t, tentacle.

The complete mating process was comprised of seven types of behaviour that were performed by males in a consecutive sequence: mate probing, shell mounting, shell circling, positioning, insemination posture, sheath withdrawal and dismounting (Fig. 3). However, when considering both male and female behaviour, the mating sequence was variable amongst different mating events. The mating sequence in successful mating events (n = 20) could be divided into four phases: pre-courtship, courtship, copulation and post-copulation. Pre-courtship phase was the period after two snails were introduced into the mating aquarium until their first contact. The first contact between the male and female could occur either by the male approaching the female or the female approaching the male. In many of the first contacts by the male (53/56 occurrences), the male moved in a courtship behaviour, such as shell mounting and shell circling. For this reason, the first contact by the male was considered as mate probing. In contrast, the first contacts by the female did not generally continue on to further behavioural stages for mating. Those females physically contacted the males and then moved away.

After the first contact that led to mating, the male performed courtship behaviour, such as touching by tentacles, mounting the female’s shell and then crawling on it in a clockwise- or counter-clockwise direction (shell circling) and adhering at the right side of the rim of the female’s last whorl. In most cases (34/37), males dismounted from the female shell after the female responded to the male’s mate probing and shell mounting by strongly retracting her cephalopodium into shell and remaining tightly adhered to aquarium. The male then repeated the courtship process again after dismounting. In the cases (20/62) where females did not respond to the male’s courtship behaviour (mate probing, shell mounting and shell circling) by a strong retraction, the male positioned himself at the right side of female’s last whorl by firmly adhering to the female’s shell, moving slightly forward until the rim of the male’s aperture overlapped with the rim of female’s aperture and then moved slightly in a left or right direction in order to locate the female gonopore and the proper position to start inserting the penis sheath into the female gonopore.

The male positioning behaviour resulted in a strong retraction by the female; however, the male still remained firmly adhered on her shell with a little movement of the foot and tentacles. After a strong retraction in response to the male’s positioning, the female then relaxed and moved or directly entered the passive stage.

The copulation phase began after the male finished the above described positioning and started penis sheath intromission. The insertion of the penis sheath could not be observed on the video recordings as the rim of the male’s aperture overlapped and covered the rim of the female’s aperture and so completely concealed the process of penis sheath intromission. However, the beginning of copulation phase was recognised, based on: (i) the typical movement of the male in the passive stage with the coiled tentacle and no movement of the foot and (ii) the movement of the female showing a strong retraction once again before ceasing all movements, entering the being in the passive stage stage.

After those activities, the male transferred semen to the female. Although semen transfer could not be directly observed, it was confirmed by the spawning of the female afterwards (n = 8) and so this posture of the mating pairs was defined as the insemination posture (following Burela and Martín (2009)). During the period that mating pairs were in the insemination posture, males ceased almost all movements, except for moving the siphon for breathing when the females stayed near the water surface, whereas females ceased movements after the strong retraction in response to penis sheath intromission, then entered the passive stage and started crawling, respectively. Generally, females crawled from the bottom of the aquarium to the water surface for gas exchange. In the insemination posture stage, females commonly performed swinging and/or jerking several times, simultaneously with passive or crawling.

After several hours, the male stopped semen transfer and withdrew his penis sheath. Although penis sheath withdrawal could not be directly observed, it was recognised, based on the movements of the foot and tentacles and detachment of the male from the insemination posture. After penis sheath withdrawal, the mating pair entered the post-copulation phase. In some cases (9/20), the male immediately dismounted from the female’s shell and moved away, but in other cases, the male remained mounted and crawled on the female’s shell before dismounting later.

**Figure 4.**
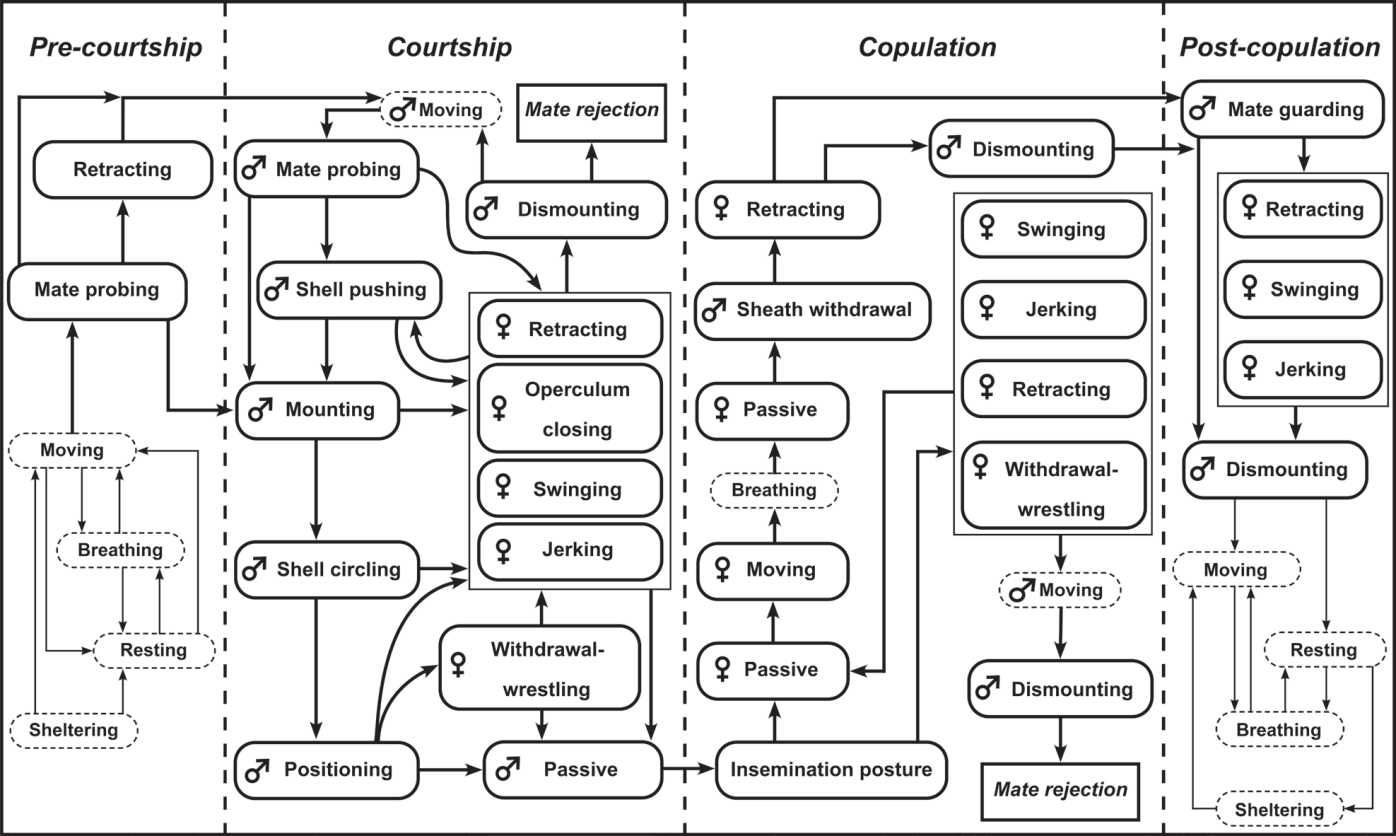
Mating sequence of *P.virescens*.

An incomplete mating process was generally caused by the female rejecting the male’s mating attempts during courting and penis sheath intromission. Although males repeated similar courtship behaviour to those performed in the complete mating process several times, some females (42/62) showed a strong reaction in rejecting mating attempts from males. Five types of mating rejection behaviour: jerking, operculum closing, retracting, swinging and withdrawal-wrestling (see description in Table 1) were identified in this study. In order to reject mating attempts by the male, the female performed various schemes of mate rejection, as shown in Fig. 4.

Mating duration was variable amongst different mating pairs, ranging from 271–840.28 minutes (n = 20; average of 607.5 ± 127.1 minutes. The mean duration of each mating phase is detailed in Table 2.

**Table 2.** Durations of mating [mean ± SD (minimum – maximum), in minutes].

Groups	All	Mating with spawning	Mating without spawning
**n**	20	8	12
**Pre-courtship**	22.3 ± 33.7 (1.1–159.4)	11.9 ± 9.5	29.2 ± 42.0
**Courtship**	145.9 ± 79.1 (35.0–281.3)	179.4 ± 82.1	123.6 ± 71.8
**Copulation**	434.3 ± 108.3 (179.0–658.0)	394.8 ± 43.7	460.7 ± 131.7
**Post-copulation**	4.5 ± 5.5 (0.0–19.6)	5.5 ± 5.9	3.9 ± 5.3
**Total**	607.5 ± 127.1 (271.0–840.3)	592.0 ± 97.0	617.8 ± 147.1

### ﻿Egg-laying behaviour

**Figure 5.**
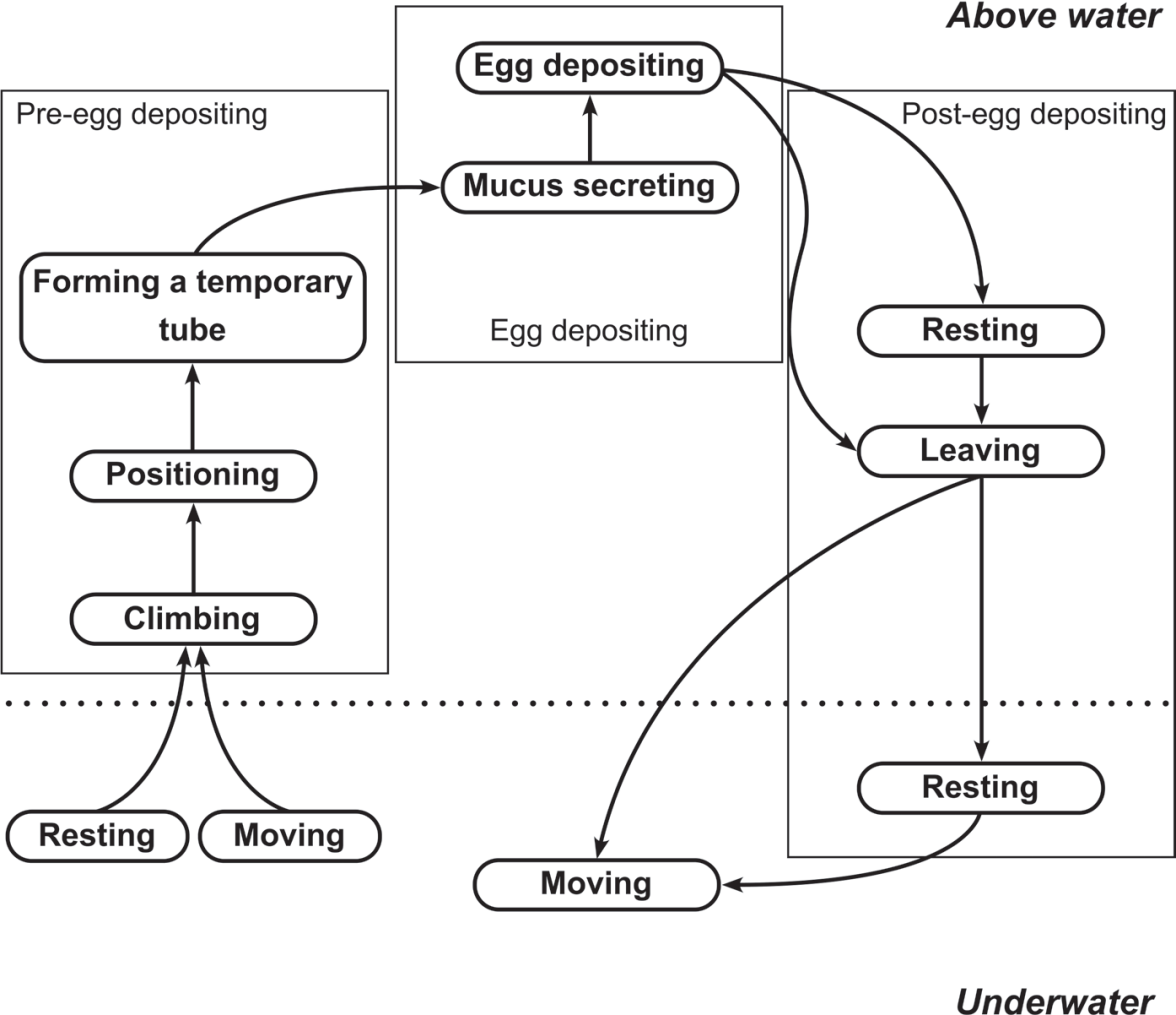
Egg-laying sequence of *P.virescens*. Rounded boxes represent behaviour performed by female *P.virescens*. Straight boxes represent egg-laying phases. Arrows indicate sequences of behaviour during egg-laying process.

Oviposition occurred in both the day and night. Egg masses were deposited on the aquarium wall above the water surface. *Pilavirescens* females performed similar sequences of egg-laying behaviour (Fig. 5), which could be divided into three phases: pre-egg depositing, egg depositing and post-egg depositing. Seven types of behaviour related to the egg-laying process were identified (Table 3): three, two and two for pre-egg depositing, egg depositing and post-egg depositing, respectively.

**Table 3.** Ethogram of egg-laying behaviour.

Behaviour	Code	Description
**Pre-egg depositing**		
Climbing	Cb	Female moves on aquarium wall above water.
Positioning	Pt	Contracts foot from all sides; tightly adheres to aquarium; foot becomes oval or heart-shaped.
Forming a temporary tube	Ftt	Right edge of foot folds ventrally into the central part of the ventral foot surface becoming a tube.
**Egg depositing**		
Mucus secreting	Ms	Secrets white mucus from gonopore; transports it to aquarium wall and smears it on aquarium wall.
Egg depositing	Ed	Lays eggs in egg mass on aquarium wall.
**Post-egg depositing**		
Leaving	Lv	Leaves egg mass by dropping or slow sliding backwards to water.
Resting	Rt	Adheres to aquarium wall with slow movement of tentacles or floats at water surface with slow movement of foot and tentacles

Pre-egg depositing began when the female climbed above the water to find an area for laying eggs, followed by adhering to the aquarium wall and forming a temporary tube that was used for transference of eggs from the female gonopore on to the aquarium wall.

After formation of the temporary tube was completed, the female entered the egg-depositing phase. The female began secreting white mucus and smeared it on the aquarium wall under her ventral foot surface and then began depositing eggs on this smeared part of the aquarium wall. Eggs appeared at the opening of female gonopore and were transported through the temporary tube to the aquarium wall near the middle of the ventral foot surface initially and finally to the top-left edge of ventral foot surface. Eggs were deposited one by one to form this egg mass. The arrangement of eggs in the egg mass started from the top-left edge to the middle and bottom edge of the foot surface, respectively and, finally, the female moved slowly backwards, while still depositing eggs to increase the available space for depositing eggs (the egg mass was normally larger than the area that the foot surface could cover and so the female had to move backwards to increase the available egg-depositing area).

**Figure 6.**
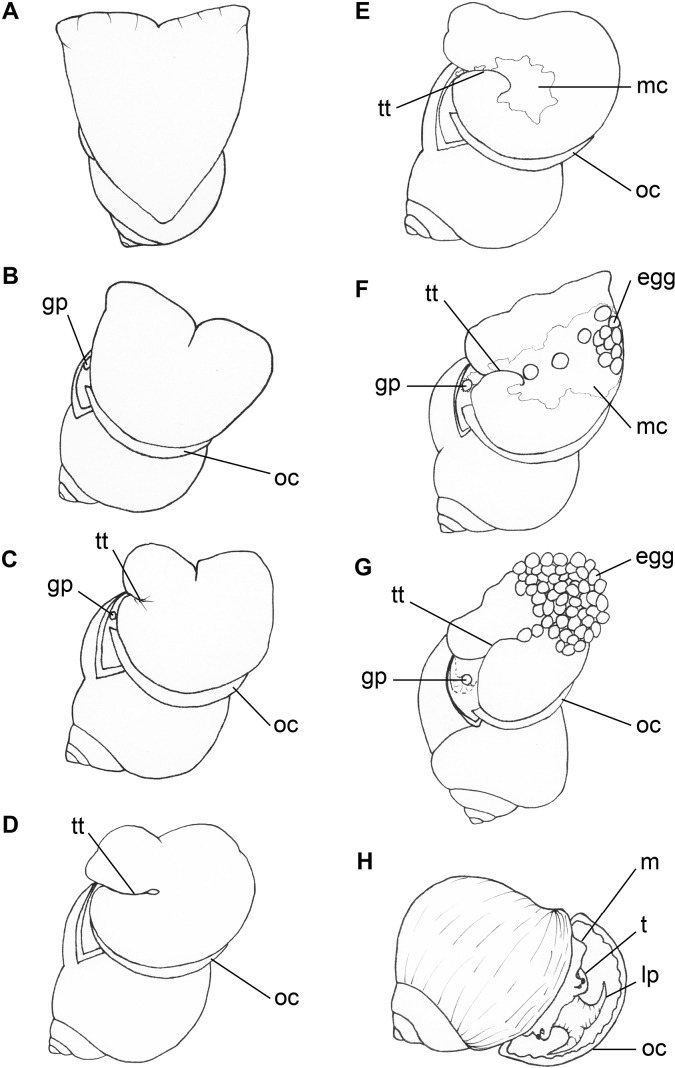
Schemes of egg-laying behaviour in *P.virescens***A** climbing **B** positioning **C, D** forming a temporary tube **E** mucus secreting **F, G** egg depositing **H** resting. Abbreviations: gp, genital pore; lp, labial palps; m, mantle; mc, mucus; oc, operculum; t, tentacle; tt, temporary tube.

After depositing the last egg, females entered the post-egg-depositing phase by slowly moving backwards into the water (n = 7) or immediately detaching from the egg mass and dropping into the water (n = 3). Most of females (n = 9) then entered the resting stage by adhering to the aquarium wall with a slow movement of the tentacles or floating at the water surface with a slow movement of foot and tentacles. There was only one female that did not enter the resting stage, but immediately started feeding on lettuce. Schematic drawing of egg-laying behaviour is provided in Fig. 6.

The overall duration of the egg-laying process (since the female left the water until she returned to the water and moved normally) was variable, ranging from 345–711 minutes (average of 483.7 ± 123.1 minutes, n = 10). Females spent the longest time in the egg-depositing phase (56.0%), followed by pre-egg depositing and post-egg depositing (23.5% and 20.5%, respectively). More details on the egg-laying duration are given in Table 4.

**Table 4.** Duration of egg laying in minutes (n = 10).

Egg-laying phases	Behaviour	Mean ± SD	Minimum – Maximum
**Pre-egg depositing**	**Climbing**	84.8 ± 52.0	26.0–175.0
	**Positioning**	14.9 ± 14.2	2.0–47.0
	**Forming a temporary tube**	13.9 ± 6.6	8.0–30.0
	**Total**	113.6 ± 55.8	38.0–198.0
**Egg depositing**	**Mucous secreting**	17.9 ± 6.7	10.0–31.0
	**Egg depositing**	253.2 ± 104.1	121.0–408.0
	**Total**	271.1 ± 102.2	131.0–421.0
**Post-egg depositing**	**Leaving**	19.4 ± 24.3	1.0–71.0
	**Resting**	75.8 ± 83.1	0.0–233.0
	**Total**	99.0 ± 93.9	9.0–252.0
**Total egg-laying period**		483.7 ± 123.1	345.0–711.0

## ﻿Discussion

Based on the video recording analysis of 62 isolated mating pairs and 10 egg-laying occasions, detailed ethograms of the mating behaviour (Table 1) and egg-laying behaviour (Table 3) of the Southeast Asian apple snail, *P.virescens*, were extracted. These ethograms allowed us to identify the sequences of mating and egg-laying behaviour of *P.virescens* leading to an improved understanding of the reproductive process of this species. The results of the current study indicated high variation in the sequence of mating behaviour of *P.virescens* and revealed its previously unknown unique egg-laying sequence.

The mating process of *P.virescens* was classified into two types following the success of the male-mating attempt: a complete and an incomplete mating process. The complete mating process was divided into four phases: pre-courtship, courtship, copulation and post-copulation, similar to the mating process that was previously reported for the golden apple snail, *Pomaceacanaliculata* (Burela and Martín 2009). The overall mating pattern: male approached female, mounted on the female shell, crawled on the female shell, adhered on the right side of the female’s last whorl in order to mate and dismounted, respectively, follows the general mating pattern described in other apple snails (Bahl 1928; Panha 1985; Burela and Martín 2009; Tiecher et al. 2014; Hayes et al. 2015; Gurovich et al. 2017); however, details of the mating behaviour performed in each mating phase were variable.

During the pre-courtship phase, two snails were introduced into the mating aquarium and so the first behaviour observed in this study was sheltering as a result of human manipulation. They sank to the bottom of the mating aquarium at random positions, but began moving shortly afterwards by slowly opening their aperture, extruding their tentacles and cephalopodium and then walked around. In most cases (40/62), snails moved directly to the water surface in order to breath. After that, the first contact between two snails happened while they were moving around in the aquarium. However, in a few cases (12/62), the snails sunk into positions which were close to each other. The first contact occurred between them immediately after they began moving. Consequently, the pre-courtship phase in this study does not represent mate-searching behaviour which may exist in *P.virescens*, as has been previously reported in the freshwater snails *Pomaceacanaliculata* (Takeichi et al. 2007) and *Biomphalariaglabrata* (Trigwell et al. 1997). However, the result indicated that males very actively performed mating behaviour. Many males (53/62) performed mate probing after the first contact. This was commonly followed by courtship behaviour, such as shell mounting and shell circling.

The courtship phase began shortly after two snails were introduced into the mating aquarium following their first contact. The male mounted the female’s shell at inconsistent positions and crawled on it in either a clockwise- or counter-clockwise direction. Clockwise shell circling was frequently observed (198/327). The positioning and insemination posture (copulation phase) was mostly observed after shell circling in the clockwise direction. This is in contrast to previous reports in other freshwater snails that male frequently performed counter-clockwise shell circling (De Boer et al. 1996; Burela and Martín 2009). It has been assumed that shell circling is an assessment of the partner size in *Pomaceacanaliculata* (Burela and Martín 2009) and *Lymnaeastagnalis*, (Koene et al. 2007). We are unable to verify this assumption as we did not examine the relationship between snail size and mating success. However, another or additional possibility is that males may perform shell circling in order to locate the position of the female gonopore as we noticed that males started circling from different initial locations on the female’s shell, but stopped circling at the right side of female’s last whorl where they could begin positioning and enter the copulation phase. In addition, more importantly, all occurrences of positioning behaviour observed in this study were performed after shell circling. This indicates that it is necessary for the male of *P.virescens* to perform shell circling for mating success.

The movement of the male snail on the female snail’s shell in order to reach the female gonopore has also been reported in *Physaacuta* (Ohbayashi-Hodoki et al. 2004) and *Lymnaeastagnalis* (Barraud 1957). Positioning took place when the male reached the right side of the female’s last whorl. The male adhered on the right side close to the rim of the female’s last whorl and then contracted its foot, slowly coiled its tentacles and moved slightly forward until its aperture overlapped with the female’s aperture. In the absence of mating rejection by the female, the pair then entered the copulation phase.

Mating rejection in apple snails was previously known only in *Pomaceacanaliculata*. Burela and Martín (2009) reported three types of mate rejection behaviour in *Pomaceacanaliculata* females: swinging, withdrawal-wrestling and operculum closing. In the same way, we also found these types of behaviour in *P.virescens*. In addition, we newly found two other types of behaviour, jerking and retracting, which could have prevented the mating process in *P.virescens*. Retracting was the strongest mate rejection observed in this study, as in most of the mating pairs, the courtship behaviour by a male ended after the female’s retraction without continuing to the copulation phase. However, retracting includes the two types of general retracting and strong retracting. General retracting is the retraction of a snail when it was touched by another snail and released soon after, which is possibly a common behaviour in this species in response to physical stimulus because it was commonly observed in both sexes when touching each other and it never led to cessation of mating behaviour. In contrast, in the case of strong retracting, retraction continued when it was touched by another snail until the two snails were separated from each other. This strong retraction was generally performed by the female and stopped mating attempts by the male in almost all cases (252/277). Similarly, different types of retraction have been reported in *Lymnaeastagnalis*: general retraction in response to disturbances and violent retraction of the cephalic part in response to penis sheath intromission that then disrupted the intromission of penis sheath in this species (Barraud 1957).

Withdrawal-wrestling and operculum closing are other strong rejections that stop mating in almost all the cases in which females showed these types of behaviour. Jerking and swinging were observed in many mating pairs and, frequently, both types of behaviour were performed. Although swinging was previously reported as a type of mate rejection behaviour in several freshwater snails (DeWitt 1991; Facon et al. 2006; Burela and Martín 2009; Tiecher et al. 2014), swinging in *P.virescens* resulted in mating failure in only a few couples. Likewise, jerking was reported as a type of mate rejection behaviour in *Physagyrina* (DeWitt 1991).

Thus, mate rejection in *P.virescens* can be categorised as two levels: (i) strong mating rejection: retracting, operculum closing and withdrawal-wrestling and (ii) weak mating rejection: jerking and swinging. Most of the incomplete mating attempts that ended in the courtship phase and resulted from the female’s mate rejection behaviour, mainly by retracting or operculum closing and, to a lesser extent, by jerking, swinging or both. There were only a few cases (6/42) of female rejection of the male during the copulation phase. In the early stage of the copulation phase, females performed retracting, jerking, swinging and withdrawal-wrestling behaviour. Five females performed withdrawal-wrestling behaviour. Four of those ceased the mating sequence, whereas only two of 19 females that performed jerking and/or swinging behaviour ceased the mating sequence.

**Figure 7.**
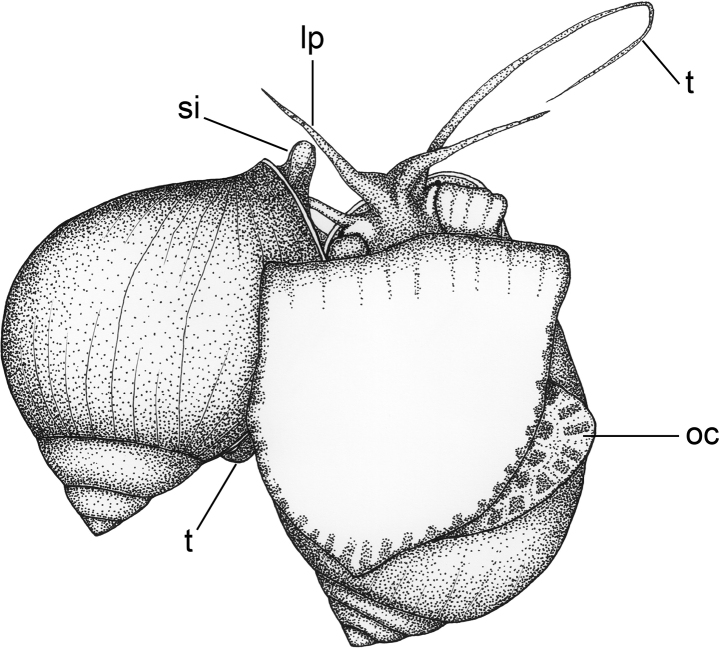
Insemination posture of *P.virescens.* Abbreviations: lp, labial palps; oc, operculum; si, siphon; t, tentacle.

Copulation began after the male positioned itself on the right side of female’s shell and then introduced the penis sheath into the female gonopore. The sheath intromission process could not be observed in this study as the male mounted on the rim of female’s shell above her aperture that overlapped with the male aperture (Fig. 7). Thus, the part of the male shell which overlapped with the female aperture completely obscured the process of penis sheath intromission. Although males mounted at the right side of the female’s last whorl above the opening of the female gonopore, similar to that described in other apple snails, such as *Asolenepulchella* (Tiecher et al. 2014), *P.globosa* (Bahl 1928), *Pomaceacanaliculata* (Andrews 1964; Albrecht et al. 1996; Burela and Martín 2009) and *Pomaceaamericanista* (Gurovich et al. 2017), their penis sheath was invisible throughout the copulation phase, whereas the penis sheath of other apple snails was visible. Thus, the insemination posture of *P.virescens* is possibly different from that in other apple snails. Although penis sheath intromission could not be seen, we recognised the copulation phase, based on the combination of the male insemination posture and the female reaction in response to penis sheath intromission – a few minutes after the male took the insemination posture, the female showed a strong retraction followed by releasing and entering the passive stage. This behavioural procedure occurred in all mating pairs from which females subsequently laid eggs. It is, thus, reasonable to assume that this repertoire is a response to penis sheath intromission.

During copulation, the male was passive, without movement, adhered firmly on the female’s shell with coiled tentacles and moved only its siphon for breathing, similar to the type of behaviour reported in other freshwater snails (Burela and Martín 2009; Soldatenko and Petrov 2012; Tiecher et al. 2014; Gurovich et al. 2017). In contrast to males, females performed various types of behaviour, such as passive, moving, jerking and swinging. Females spent most of the time during copulation in a passive state. They could move around in the mating aquarium. However, in almost all cases, those movements were to crawl up to the water surface in order to breath. Jerking and swinging behaviour performed in copulation phase was indistinguishable actions from those that rejected courtship in some cases. Burela and Martín (2009) reported swinging in *Pomaceacanaliculata* only during courtship and early copulation (penis sheath intromission). We observed that some *P.virescens* females performed jerking and/or swinging several times throughout the copulation period while they were passive or moving. At the end of copulation, the male withdrew the penis sheath from the female gonopore, which we recognised, based on the movements of the male combined with the female’s retracting.

After penis sheath withdrawal, some males (11/20) still remained mounted on the female’s shell and crawled or passively adhered on to it for few minutes before dismounting, whereas other males immediately dismounted from the female’s shell. The behaviour of males that continued to adhere on to the female’s shell after penis sheath withdrawal was described as mate guarding behaviour in *Pomaceacanaliculata* (Burela and Martín 2011).

Oviposition took place several days after copulation, but was widely variable, ranging from 4–51 days (average 14 ± 15 days, n = 10), which is much longer than that within the 1 or 2 days after copulation reported in *P.globosa* (Bahl 1928). Females laid white spherical eggs in one mass on the aquarium wall at various positions, but mostly near the water surface. In nature, *P.virescens* lays eggs on various substrates (e.g. on the ground, floating wood and plants) away from the water (Semper 1862; Ng et al. 2020). The average clutch size was 104.3 ± 54.0 eggs (range from 50–205 eggs, n = 10), which is similar to clutch sizes of 70–80 and 100–150 eggs per mass collected from natural habitats (Semper 1862; Ng et al. 2020).

Oviposition is known to be frequent during the night or early morning in *Pomaceacanaliculata* (Albrecht et al. 1996; Heiler et al. 2008; Gilal et al. 2015), *Pomaceaamericanista* (Gurovich et al. 2017) and *P.globosa* (Bahl 1928). Nocturnal oviposition was suggested to be an avoidance from predators, heat and desiccation (Estebenet and Martín 2002; Gurovich et al. 2017). Oviposition of *P.virescens* occurred during both the day and night in this study conducted indoors. It is possible that different conditions may be the reason for the observed difference in *P.virescens*; however, further research would be necessary to explore the differences. In addition, we observed that *P.virescens* in collective tanks in outdoor conditions frequently laid eggs after rain. This suggests that *P.virescens* may naturally lay eggs after rain as an alternative means to avoid heat and desiccation. This could similarly be the case in other apple snails that have been known to lay eggs typically in the night-time.

The egg-laying behavioural process of *P.virescens* is similar to that of *P.globosa* (Bahl 1928). However, *P.virescens* was never observed to dig a hole to deposit its eggs as was reported in *P.globosa*. In addition, several new types of behaviour were identified and reported for the first time in this study. The egg-laying process was comprised of seven major types of behaviour (Table 3), which could be split into the three phases of pre-egg depositing, egg depositing and post-egg depositing. The first sign of oviposition is climbing. In the same manner as other aerial egg-laying apple snails (genus *Pomacea* and *Pila*) (Bahl 1928; Andrews 1964; Cowie 2002; Gurovich et al. 2017), *P.virescens* females move above the water to lay eggs. It was reported that *P.globosa* forms the foot as a dome shape to arrange eggs in a compact mass (Bahl 1928), whereas we found that *P.virescens* contracts the entire foot forming an oval or heart shape (seen from the ventral view) instead of the dome shape. Subsequently, the right edge of the foot forms a tube shape used for transferring the eggs from the gonopore to the aquarium wall. Formation of this temporary egg transferring tube has also been reported in *P.globosa* (Bahl 1928). In other genera (e.g. *Pomacea* and *Asolene*), females form a nuchal groove at the right side of the dorsal foot surface for transferring the eggs from the gonopore to the substrate (Andrews 1964; Albrecht et al. 1996; Tiecher et al. 2014; Gurovich et al. 2017). The formation of an egg transferring tube in apple snails has only been reported within the genus *Pila* which may suggest the evolution of distinct egg-laying behaviour in this genus.

After forming the egg-transferring tube, instead of depositing eggs directly on the (glass) substrate, *P.virescens* secreted a smear of white milky mucus. The mucus appeared from the female gonopore and was then transported through the egg-transferring tube and became smeared on the aquarium wall by moving the ventral foot surface. This process has never been reported before in any apple snails. Eggs were laid one by one, appearing at the opening of the gonopore and transported through the egg transferring tube to the middle area of the ventral foot surface, similarly to that described in *P.globosa* (Bahl 1928). Subsequently, eggs were transported from the middle to the top-left edge of the ventral foot surface. Instead of laying flaccid eggs directly as reported in other apple snails (Bahl 1928; Andrews 1964; Albrecht et al. 1996; Gurovich et al. 2017), *P.virescens* laid up to the first 10 eggs with the milky mucus and the later eggs with little or no milky mucus. The egg mass was formed by arranging the eggs at top-left edge of the ventral foot surface and then expanding to the middle and lower edge. If egg mass grew larger than the ventral foot surface could hold, the female moved slowly backwards and simultaneously continued egg laying.

The behaviour of *P.virescens* after leaving the water until laying the last egg was consistently observed amongst egg-laying events of different females. The behaviour varied only during the post-egg-deposition phase. Two females adhered on the egg mass, one for 15 minutes and the other for 23 minutes, after laying the last egg and then left the egg mass by slowly sliding backwards into the water. Three females immediately dropped into the water after laying their last egg, while the rest (n = 5) slowly slid backwards to the water after laying the last egg. These post-egg depositing variations were also reported in *Pomaceacanaliculateta* (Albrecht et al. 1996). Resting and refreshing seem to be important for females after finishing laying eggs. Almost all the females (n = 9) entered the resting stage, possibly resulting from energy expenditure for laying eggs.

Behavioural characteristics identified in this study increase our understanding of the diversity of reproductive behaviour in a Southeast Asian apple snail. Although types of mating behaviour largely followed those previously recorded in South American confamilial snails (Burela and Martín 2009; Tiecher et al. 2014; Hayes et al. 2015; Gurovich et al. 2017), our results reveal some variations. In addition, our results on egg-laying behaviour highlight the distinct aerial egg-laying sequence in *P.virescens*. Further research, including comparative studies on reproductive behaviour of different species would provide new insights into the adaptation and evolution of the reproductive process of apple snails (Hayes et al. 2015).

## ﻿Additional information

### Author ORCIDs

Supunya Annate https://orcid.org/0009-0001-6359-7954

Ting Hui Ng https://orcid.org/0000-0002-5123-0039

Chirasak Sutcharit https://orcid.org/0000-0001-7670-9540

Somsak Panha https://orcid.org/0000-0002-4431-2458

### Data availability

All of the data that support the findings of this study are available in the main text.
